# hnRNPH2 gain-of-function mutations reveal therapeutic strategies and a role for RNA granules in neurodevelopmental disorders

**DOI:** 10.1172/JCI171499

**Published:** 2023-07-17

**Authors:** Benjamin A. Kelvington, Ted Abel

**Affiliations:** Iowa Neuroscience Institute and Department of Neuroscience and Pharmacology, Carver College of Medicine, University of Iowa, Iowa City, Iowa, USA.

## Abstract

hnRNPH2-related neurodevelopmental disorder (NDD) is caused by mutations in the *HNRNPH2* gene and is associated with substantial challenges, including developmental delay, intellectual disability, growth delay, and epilepsy. There is currently no therapeutic intervention available to those with hnRNPH2-related NDD that addresses its underlying mechanisms. In this issue of the *JCI*, Korff et al. studied specific gain-of-function mutations associated with hnRNPH2-related NDD, with the help of mouse models that recapitulate key features of the condition in humans. Their work paves the way for therapeutic approaches that aim to reduce the expression of mutant hnRNPH2 and highlights a role for disrupted RNA granules in neurodevelopmental and neurodegenerative disorders.

## hnRNPH2 mutations expose mechanisms mediating neurodevelopmental disorder–relevant phenotypes

**Figure 1:**
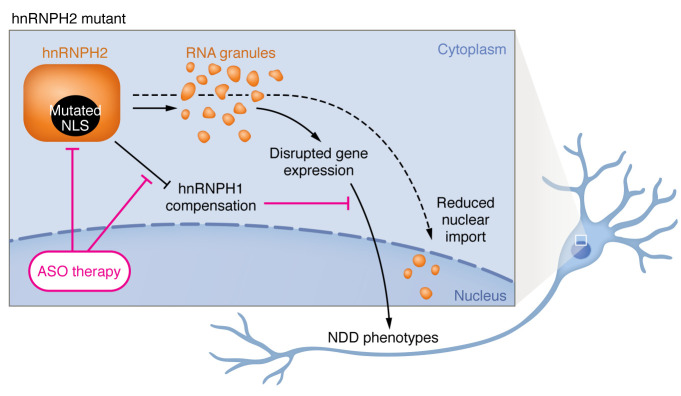
The consequences of NDD-associated mutations in hnRNPH2 include altered localization, disrupted gene expression, and NDD-relevant phenotypes. The presence of mutant hnRNPH2 inhibits genetic compensation by hnRNPH1. Reduced nuclear import of hnRNPH2 mutants leads to their incorporation into cytoplasmic RNA granules. Mutant hnRNPH2-containing RNA granules alter patterns of gene expression that ultimately lead to the phenotypes associated with hnRNPH2-related NDD. The expression of mutant hnRNPH2 may be reduced using an antisense oligonucleotide (ASO) as a therapeutic strategy.

Proteins in the heterogeneous nuclear ribonucleoprotein (hnRNP) family regulate multiple aspects of RNA biology, including RNA localization, processing, and translation. Mutations in *HNRNPH2* that disrupt the nuclear localization signal (NLS) of the hnRNPH2 protein are associated with a neurodevelopmental disorder (NDD) characterized by intellectual disability, motor and language delays, growth and musculoskeletal dysmorphias, and epilepsy (1–3). In this issue of the *JCI*, Korff et al. (4) demonstrated that mutations disrupting the NLS resulted in the accumulation of mislocalized hnRNPH2 protein within cytosolic RNA granules, structures that control RNA dynamics. The authors then examined the effect of hnRNPH2 mutations in vivo by generating two mutant knockin mouse lines. These mice exhibited several characteristics relevant to the human hnRNP-related NDD, including a male-specific reduction in survival and growth, craniofacial dysmorphias, seizure susceptibility, motor deficits, anxiety-like behavior, and memory deficits. Importantly, these characteristics were not observed in mice with a complete knockout of *Hnrnph2*. *Hnrnph2-*knockout animals displayed upregulation of the closely related *Hnrnph1* transcript, indicating a compensatory mechanism between hnRNP family members. These results suggest that the characteristics of hnRNPH2-related NDD are associated with a gain of function of the hnRNPH2 protein, likely related to mislocalization of mutant hnRNPH2 to cytoplasmic RNA granules (Figure 1). Korff et al. (4) has considerable therapeutic implications, as their findings suggest that targeting expression of mutant *HNRNPH2*, such as with an antisense oligonucleotide, may induce compensatory expression of *HNRNPH1* and consequently ameliorate the symptoms of hnRNPH2-related NDD. This work also highlights the translational importance of characterizing specific syndrome-related alleles in mouse models that best reproduce the molecular mechanisms mediating NDD phenotypes in human hnRNPH2-related NDD. The results also underscore the idea that NDD-associated genes should not be assumed to act through a simple loss-of-function mechanism and, thus, cannot always be adequately studied via knockout models. Rather, it is imperative to investigate specific NDD-associated mutations to determine their precise consequences for gene function and the downstream mechanisms mediating NDD phenotypes.

## Investigating functions of RNA granules in NDDs

Beyond the substantial therapeutic implications, Korff et al. (4) introduce powerful mouse models to study how the disruption of RNA granules contributes to NDDs. A growing body of evidence suggests that the genetic contributions to complex NDDs such as autism spectrum disorder converge on molecular pathways that regulate gene expression (5–7). Furthermore, recent evidence suggests that the disruption of genes involved in RNA granule formation is associated with a subset of NDDs (8). Therefore, it is likely that disrupting the regulation of gene expression is a crucial consequence of NDD-associated mutations in RNA granule genes, including hnRNPH2 (Figure 1). However, while RNA granules are known to include subtypes that vary by composition and disease associations, including stress granules and activity-dependent granules, little is known about the molecular mechanisms by which RNA granules mediate gene expression changes that contribute to NDDs. The work by Korff et al. (4) suggests that mutations in hnRNP family members may provide vital insights into these mechanisms. hnRNP family members have been implicated in an array of NDDs (9). Like the outcomes observed in hnRNPH2-related NDD, mutations within the NLS of the closely related hnRNPH1 protein are also associated with NDDs, indicating that the underlying molecular processes mediating NDD phenotypes may be shared across the family (10, 11). Other NDD-related genes also function in RNA granules and disrupt gene expression. For example, in fragile X syndrome, the fragile X protein is known to incorporate into activity-dependent RNA granules, alongside hnRNPH family members, to regulate activity-dependent mRNA translation (12, 13).

The hnRNPH2-knockin mutant mice generated by Korff et al. (4) create the opportunity to address crucial questions regarding the molecular mechanisms underlying the impact of RNA granules in NDDs. For example, how do RNA granules regulate activity-dependent gene expression? And how does disruption of this function contribute to NDDs? Additionally, how do these changes in gene expression compare with other NDD models? Korff et al. begin to assess gene expression in their model by performing RNA-Seq in induced pluripotent stem cell–derived neurons and cortical tissue from mutant mice. Both data sets suggest that splicing variants play a critical role in *HNRNPH2* mutant-associated changes in gene regulation. Thus, the phenotypes associated with hnRNPH2-related NDD may not be caused by simple upregulation or downregulation of genes but rather the production of alternative protein products with distinct functions. Furthermore, the sequencing data in mice revealed upregulation of the putative NDD-related genes *CTNNA2*, *TNPO2*, *ASH1L*, and *SHANK1*, which highlights potential convergent pathways connecting hnRNPH2-related NDD with other syndromic NDDs. These models pave the way for the investigation of how *HNRNPH2* mutations act through RNA granules to disrupt activity-dependent gene expression in a brain region–specific and cell-type–specific manner. Furthermore, because RNPs play a role in various aspects of RNA biology, and mutant hnRNPH2 localizes to RNA granules outside the nucleus, the full effect of these mutations on gene expression may not be captured by traditional RNA-Seq. Translatome and proteome analyses will be necessary to gain a more complete picture of gene regulation in this model. Protein-level analyses of purified RNA granules will also help to uncover the physical interactions mediating RNA granule disruption in NDD-associated hnRNPH2 mutants.

## RNA granules connect neurodevelopmental and neurodegenerative disorders

Studying disruptions in hnRNP proteins and their downstream consequences in RNA granules may also provide crucial insights into the mechanisms shared between NDDs and neurodegenerative disorders. This connection has long been understudied but has recently come into focus, as emphasis has been placed on supporting individuals with NDDs throughout the life span. The hnNRP family has been implicated in a variety of neurodegenerative conditions (14). For example, mutations in *HNRNPA1* confer risk of developing multiple neurodegenerative disorders, including amyotrophic lateral sclerosis and frontotemporal lobar degeneration, and pathogenic mutations disrupting the NLS of the HNRNPA1 protein result in disrupted nucleocytoplasmic transport and altered RNA granule dynamics (15, 16). Additionally, dysregulation of mRNA splicing contributes to neurodegenerative disorders associated with hnRNPA1, such as amyotrophic lateral sclerosis, frontotemporal lobar degeneration, Alzheimer’s disease, and spinal muscular atrophy (17). These mechanisms mirror those proposed by Korff et al. (4) and suggest that convergent mechanisms among hnRNP family members may underlie both neurodevelopmental outcomes and neurodegenerative ones. Furthermore, premutations in the *FMR1* gene that do not cause fragile X syndrome can result in a neurodegenerative disorder known as fragile X-associated tremor/ataxia syndrome, indicating that neurodegenerative mechanisms may be shared by mutations in RNA granule genes extending beyond the hnRNP family (18, 19).

The mouse models generated by Korff et al. (4) provide substantial opportunities for investigation of the mechanisms connecting NDDs with neurodegenerative disorders. Although the neurodegenerative consequences specific to hnRNPH2-related NDD are unknown, the condition has yet to be studied in any individual older than 38 years of age (2). hnRNPH2 may be an especially promising target to study these mechanisms, as the authors show that *HNRNPH2* expression persists in the brain throughout aging, even as *HNRNPH1* levels wane. Thus, the detrimental impact of *HNRNPH2* mutations may escalate with age. Although P209L mutants exhibit markedly reduced survival, studies of female P209L mice as well as R206W mutants of both sexes have the potential to answer many questions about the role of *HNRNPH2* mutations in aging. For example, how do the effects of hnRNPH2 mutants on gene expression change as mice age? And how do mutations in *HNRNPH2* affect RNA granule dynamics and the aggregation of proteins implicated in neurodegeneration? Additionally, how do the phenotypes observed in aged hnRNPH2 mutants compare with other models of neurodegenerative disorders? Answers to these questions will begin to define the potential role of *HNRNPH2* mutations in aging and neurodegeneration. They will also provide critical insight into the mechanistic connections between neurodevelopmental and neurodegenerative disorders. Knowledge of these connections could be greatly strengthened by the generation of mouse models specific for neurodegeneration-associated mutations in hnRNP family members, like the NLS mutations observed in *HNRNPA1*. These mice would create the opportunity for intensive study of the mechanisms by which similar disruptions in hnRNP family members contribute to both neurodevelopmental and neurodegenerative disorder.

Korff et al. (4) demonstrate the value of generating mouse lines that model specific NDD-associated mutations in genes that expose the precise molecular mechanisms mediating NDD phenotypes. They reveal that mutations disrupting the NLS of the hnRNPH2 protein result in NDD-relevant phenotypes that are not observed in *HNRNPH2-*knockout animals. Uncovering this mechanism paves the way for the investigation of therapeutic approaches that target mutant *HNRNPH2* transcripts, induce compensatory *HNRNPH1* expression, and ultimately alleviate the symptoms of HNRNPH2-associated NDD. Further study of these mouse models, as well as continued use of this strategy to generate additional models, will provide much needed information regarding the molecular underpinnings of and, ultimately therapeutic interventions for, disorders of the brain spanning from development to degeneration.

## Version 1 07/17/2023

Electronic publication
